# Characterising the patterns of and factors associated with increased alcohol consumption since COVID‐19 in a UK sample

**DOI:** 10.1111/dar.13256

**Published:** 2021-03-03

**Authors:** Melissa Oldham, Claire Garnett, Jamie Brown, Dimitra Kale, Lion Shahab, Aleksandra Herbec

**Affiliations:** ^1^ Department of Behavioural Science and Health University College London London UK

**Keywords:** COVID‐19, alcohol, drinking pattern, change, United Kingdom

## Abstract

**Introduction:**

To examine changes in drinking patterns and to assess factors associated with reported increases in frequency of drinking, units consumed and frequency of heavy episodic drinking (HED) during the UK lockdown.

**Methods:**

Online cross‐sectional survey of 2777 self‐selected UK adults.

**Results:**

Thirty percent of participants reported drinking more frequently in lockdown, 16% reported drinking more units per drinking occasion and 14% reported more frequent HED. For men and women, increased frequency of drinking was associated with being less likely to believe alcohol drinking would lead to greater chance of catching COVID‐19 (men: OR = 0.99, 95% CI = 0.98, 1.00; women: OR = 0.99, 95% CI = 0.99, 1.00) and deterioration in psychological wellbeing (OR = 1.27, 95% CI = 1.04, 1.54; OR = 1.29, 95% CI = 1.11, 1.51); increased unit consumption was associated with deterioration in financial situation (OR = 1.50, 95% CI = 1.21, 1.86; OR = 1.31, 95% CI = 1.05, 1.64) and physical health (OR = 1.31, 95% CI = 1.03, 1.67; OR = 1.66, 95% CI = 1.31, 2.10). Finally, increases in the frequency of HED were associated with deterioration in psychological wellbeing (OR = 1.65, 95% CI = 1.25, 2.18; OR = 1.46, 95% CI = 1.17, 1.82) and being furloughed (OR = 3.25, 95% CI = 1.80, 5.86; OR = 2.06, 95% CI = 1.19, 3.56). Other gender differences were detected, for example, living with children was associated with an increase in units consumed (OR = 1.72, 95% CI = 1.09, 2.73) and the frequency of HED (OR = 2.40, 95% CI = 1.44, 3.99) for men, but not women.

**Discussion and Conclusions:**

In this self‐selected UK sample, a significant proportion of individuals reported drinking more frequently in lockdown, drinking more units per drinking occasion and more frequent HED. There were consistent predictors of increased consumption across men and women, but other gender differences were detected. This study identifies groups that may require targeted support in future lockdowns.

## Introduction

COVID‐19 is a respiratory disease that was declared a global pandemic in March 2020. Many countries introduced social distancing measures in order to control the spread of the virus. In the UK, a national ‘lockdown’ was introduced on 24 March 2020, requiring significant disruption to people's lives. Lockdown had a polarising impact on drinking patterns in the UK [1, 2, 3, 4], with 26% of drinkers drinking less and 26% drinking more [3]. However, it is currently unclear how increases in alcohol are characterised in relation to drinking patterns and whether particular groups are more at risk of increased drinking. Increases in alcohol consumption often have negative public health and social consequences [5, 6, 7, 8, 9, 10, 11], particularly if driven by individuals at higher risk of harm. In July 2020, the first peak of infections and mortality in the UK passed and lockdown policies were relaxed. However, as of November the second wave epidemiologists predicted [12, 13] seems to be underway and a national lockdown was reintroduced. Identifying the drinking practices and groups more susceptible to increases in alcohol consumption during lockdown could inform the targeting of public health messaging and the provision of support for alcohol reduction during future lockdowns.

Early data have identified some characteristics associated with increases in alcohol consumption in lockdown. Increases in consumption are more likely amongst heavier drinkers [1], this is particularly concerning as heavier drinkers are more susceptible to alcohol‐related harms [14]. Similarly, being younger, female, having post‐16 education qualifications, having an annual household income over £30 000, being significantly stressed about catching or becoming seriously ill from COVID‐19, being significantly stressed about finances and having a diagnosed anxiety disorder were all associated with increased drinking in lockdown [3], as was being furloughed from work [15]. However, these studies looked at overall increases in consumption and did not differentiate between different patterns of drinking.

It is important to consider increases in alcohol consumption in relation to drinking patterns as different patterns are associated with different harm profiles [10, 14, 16]. Heavy episodic drinking (HED), defined as drinking more than 6 units on one occasion, is typically associated with greater risk of hospitalisation, hazardous and delinquent behaviours and alcohol‐related harms [10, 14, 16]. Therefore, increases in the frequency of heavy episodic drinking in particular may carry particularly negative consequences. Here, for the first time we characterise the nature of increases in alcohol consumption during COVID‐19‐related lockdown in the UK. Specifically, we will separately examine increases in the: (i) frequency of drinking occasions; (ii) units consumed on drinking occasions; and (iii) increases in HED occasions, and the factors that are associated with them.

It is particularly important to examine the socio‐demographic characteristics associated with each of these factors as we may expect to see socio‐demographic differences in patterns of consumption and subsequent alcohol‐related harms. Being male [17, 18] and younger [18] is associated with an increased likelihood of HED. Furthermore, drinkers who drink more heavily [19], are more deprived [20, 21] and are younger [10, 22] are more likely to experience alcohol‐related harms. Different socio‐demographic groups may also have been differentially affected by the lockdown. For example, women were more likely than men to lose their jobs and have also disproportionately carried the burdens of increased child care and housework [23, 24]. This, coupled with increased loneliness [25], means that women have experienced greater declines in wellbeing [25, 26, 27]. Furthermore, more deprived groups may have had more serious financial concerns and may have been isolated in less comfortable accommodation. It is important to consider the socio‐demographic characteristics of those drinking more in lockdown, particularly in relation to HED, in order to monitor the possibility of emerging or increased inequalities.

Lockdown has also caused stress about work, health, finances and caring responsibilities [27]. Stress can have a polarising effect on drinking with some responding maladaptively by drinking heavily and others abstaining altogether [28]. As such, some people experiencing stress could be more likely to engage in HED. The home environment, working status and health concerns could also impact on patterns of increased consumption in lockdown [3, 15]. Those in key worker roles may be less likely to drink more frequently, but could be more likely to engage in HED on their days off, whereas HED may be less likely amongst those responsible for caring for dependants.

Previous research has outlined interactions between alcohol consumption and other behaviours, specifically smoking [29] and exercise [30, 31]. The effect lockdown will have on these interactions is unclear. COVID‐19 specifically affects the lungs and that along with the more general focus on health as a result of the pandemic may have served as a teachable moment encouraging some to quit smoking, this is supported by early data showing an uptick in smoking quit attempts as a result of COVID‐19 [4]. It is possible that those looking to quit may drink more as they may see it as a less directly harmful coping behaviour than smoking. In the early stages of lockdown, people were encouraged to stay at home and were allowed to leave the home once a day for exercise. Early evidence shows that lockdown may have increased population interest and engagement in physical activity [32]. Alternatively, the closure of gyms and leisure centres may have led to a reduction in exercise for some. Changes in exercise could lead to changes in drinking patterns, some may use exercise to attempt to offset the potential harms of increased drinking or a reduction in exercise could lead to increased consumption as a result of boredom.

In order to inform public health messaging and the provision of support for alcohol reduction during future waves of COVID‐19 in the UK, we examine which socio‐demographic, environmental, health factors and other behaviours are associated with increases in the frequency of drinking occasions, increases in units consumed on drinking occasions and the frequency of HED occasions. As we know that there is a divergence in changes to drinking in lockdown between men and women [3] and differences in the way men and women have been affected by changes in circumstances under lockdown [23, 24], all analyses will be stratified by gender.

This study aims to answer the following research questions among adult men and women in the UK who drink separately:

1. What proportion of adult drinkers have increased, maintained and decreased the frequency of their alcohol consumption, units consumed per drinking occasion and the frequency of heavy drinking occasions during the lockdown in the UK?
2. What socio‐demographic, environmental, health factors and other behaviours are associated with:
  - increases in the frequency of drinking in lockdown?
  - increases in units consumed per drinking occasion in lockdown?
  - increases in the frequency of HED in lockdown?

## Methods

### Design and study population

The analysis plan was pre‐registered and is available at https://osf.io/vmc5u/. We analyse cross‐sectional data from the baseline wave of an ongoing retrospective longitudinal online survey of UK adults—the HEalth BEhaviours during the COVID‐19 pandemic (HEBECO) study. The eligibility criteria were that participants be aged 18 or older, be fluent in English and be willing to complete follow‐up surveys online. More information about the survey and an overview of the questions is available online (https://osf.io/sbgru/).

Our self‐selected sample of UK‐based adults (18+) completed the baseline survey of the HEBECO study between 30 April 2020 (initiation of recruitment) and 14 June 2020 (inclusive), when the strictest lockdown policies were relaxed (e.g. non‐essential shops were re‐opened). Recruitment was online and through a number of channels, including unpaid posts and paid advertisements on Facebook, Google and Reddit and through relevant mailing lists of UK universities, charities, local government, as well as through the networks of Cancer Research UK and Public Health England. To account for the non‐random nature of the sample, all data are weighted to the proportions of sex, age, ethnicity, education and country of living obtained from the Office for National Statistics [33]. For full details on the recruitment strategies and weighting for the study, visit https://osf.io/sbgru/.

### Measures

A summary of the measures is provided below, but please see Appendices S1 and S2 for a full overview of the questions asked and how they were operationalised.

#### Pre/post‐COVID‐19

Rather than relying on potentially arbitrary dates for the start of the COVID‐19 pandemic, participants were asked to identify the moment when COVID‐19 started to impact on their lives, and use that moment as an anchor for ‘before’ and ‘since’ COVID‐19 throughout the survey (referred to as ‘pre’/‘post’‐COVID‐19 throughout). Those who reported that COVID‐19 had not impacted them were asked to consider 21 March 2020 as the time since COVID‐19, in line with the UK government's introduction of strict social distancing measures and the closure of pubs.

#### Outcome variables

All outcome variables are derived from the Alcohol Use Disorders Identification Test‐Concise (AUDIT‐C), a three‐item questionnaire asking about the frequency of drinking, average units consumed on a drinking occasion and frequency of HED. As outlined above, participants responded to two sets of the AUDIT‐C questions, one in reference to the 12 months pre COVID‐19 and the second asking the same information for the period following the self‐assigned date where they believed COVID‐19 started to affect them. The three outcome variables were increase in the frequency of drinking (1 = increase, 0 = no change or drinking less), increase in units consumed per drinking occasion (1 = increase, 0 = no change or drinking less) and increase in the frequency of HED (1 = increase, 0 = no change or drinking less). An increase was defined as a higher score on the relevant AUDIT‐C item post‐COVID‐19.

#### Predictor variables

The following socio‐demographic characteristics were assessed: age (continuous in years 18–110+), ethnicity (white = 1, Black, Asian and Minority Ethnic = 0) and education (with post‐16 qualifications = 1, other = 0). Two measures of baseline drinking characteristics were included: alcohol reduction attempts in the last 12 months (continuous between 0 and 14+) and pre‐COVID‐19 AUDIT‐C score (continuous between 0 and 12). Six variables were focused on lockdown living situation: living alone (1 = yes, 0 = no), living with children (1 = yes, 0 = no), keyworker (1 = yes, 0 = no), furloughed (1 = yes, 0 = no), working from home (1 = yes, 0 = no) and overall experience of social distancing (continuous 0–100). Changes to lifestyle as a result of COVID‐19 were examined over six domains: living conditions, financial situation, psychological wellbeing, social relationships, family relationships and physical health. Participants rated each of these areas of their life pre‐ and post‐COVID‐19 on a scale of ‘1 (poor)’ to ‘5 (excellent)’. Change scores were calculated where negative score indicated an improvement. Variance Inflation Factors (VIF) were checked for multicollinearity, and no issues detected (VIFs <1.7). Health concerns were measured by two variables: number of existing health conditions (continuous between 0 and 11) and perceived alcohol risk, which measured participants beliefs that alcohol would put them at greater risk of catching or having worse outcomes from COVID‐19 (continuous 1–100); finally, smoking status (1 = current smoker, 2 = ex‐smoker and 3 = never smoker) and change in the frequency of physical activity (change score of total number of days of strength training/week and the number of sessions of aerobic exercise/week combined pre‐ and post‐COVID‐19).

#### Analyses

The protocol and analysis plan were pre‐registered on Open Science Framework (https://osf.io/pnrhq/).

Analyses were conducted using spss on full cases (missing data described in the results). All analyses were stratified by gender. All data were weighted to the proportions of sex, age, ethnicity, education and country of living obtained from the Office for National Statistics [33] to account for the non‐random sample.

Descriptive statistics were calculated to characterise the sample in terms of socio‐demographic characteristics. The proportions of those who reported increases, decreases and no change in the frequency of drinking occasions, units consumed and the frequency of HED were calculated.

Logistic regression models were conducted to examine associations of: (i) drinking more frequently post‐lockdown (vs. drinking less or the same); (ii) drinking more units per occasion post‐lockdown (vs. less or the same); and (iii) more frequent HED post‐lockdown (vs. less or the same) with: socio‐demographic factors, pre‐COVID‐19 drinking characteristics, lockdown living situation, changes to lifestyle, health concerns and other behaviours.

HEBECO study data were collected and managed using REDCap electronic data capture tools hosted at University College London [34, 35]. REDCap (Research Electronic Data Capture; https://projectredcap.org/) is a secure, web‐based software platform designed to support data capture for research studies, providing: (i) an intuitive interface for validated data capture; (ii) audit trails for tracking data manipulation and export procedures; (iii) automated export procedures for seamless data downloads to common statistical packages; and (iv) procedures for data integration and interoperability with external sources.

## Results

### Participant characteristics

A total of 2994 participants responded to this survey, 10 participants reporting that they were non‐binary and seven who preferred not to say are excluded from this analysis due to the gender stratification and low numbers in these categories. This leaves a sample of 2977 participants (weighted *n* = 2777, weighted %: 52% female, 48% male). Table 1 presents the participant characteristics for the whole sample and by gender. The majority of participants were from England (86%), with 7% from Scotland, 6% from Wales and 1% from Northern Ireland.

**Table 1:** Participant characteristics for the total sample and stratified by gender^a^

	All	Female	Male
Weighted, *n*	2777	1451	1326
Age, *M* (SD)	49.07 (16.58)	48.78 (16.12)	49.39 (17.08)
Ethnicity, % white^b^	89.40%	89.70%	89.10%
Education, % with post‐16 qualifications	67.40%	66.90%	68.00%
Baseline AUDIT‐C, scales 1–12, *M* (SD)	5.77 (3.37)	4.67 (2.93)	6.93 (3.41)

^a^ Based on weighted data.

^b^ This is due to a large majority of our sample being white, a limitation of the study that is discussed later.

AUDIT‐C, Alcohol Use Disorders Identification Test‐Concise.

### Changes in drinking

Amongst 50% of participants, there was no change in the frequency of drinking pre‐ and post‐COVID‐19, 30% of participants reported drinking more frequently and 20% reported drinking less frequently (Table 2). The majority (61%) of participants reported consuming the same number of units per drinking occasion pre‐ and post‐COVID‐19, 16% reported drinking more and 22% drinking less. Finally, 61% of participants reported no change in the frequency of HED occasions pre‐ and post‐COVID‐19, 14% reported an increase and 26% reported a decrease. The patterns of drinking for each outcome measure were roughly comparable across men and women with the most disparity in units consumed. Women were more likely to drink the same units as pre‐COVID‐19 (66% vs. 56% of men), and men were more likely to drink both more units (19% rather than 14% of women) and less units (25% relative to 20% of women).

**Table 2:** Changes in the frequency of drinking occasions, units consumed and HED occasions

	Frequency of drinking occasions			Units consumed per drinking occasion			Frequency of HED occasions		
	All (*n* = 2777^a^)	Female (*n* = 1451)	Male (*n* = 1326)	All (*n* = 2213)	Female (*n* = 1155)	Male (*n* = 1058)	All (*n* = 2250)	Female (*n* = 1178)	Male (*n* = 1072)
Drinking more post‐COVID‐19	828 (30%)	442 (31%)	386 (29%)	361 (16%)	161 (14%)	200 (19%)	306 (14%)	160 (14%)	146 (14%)
Drinking same post‐COVID‐19	1381 (50%)	733 (51%)	656 (50%)	1360 (61%)	765 (66%)	595 (56%)	1369 (61%)	731 (62%)	638 (60%)
Drinking less post‐COVID‐19	560 (20%)	276 (19%)	284 (21%)	492 (22%)	229 (20%)	263 (25%)	575 (26%)	287 (24%)	288 (27%)
Missing values^b^	0	0	0	563^b^	296	268	527^a^	273	254

^a^ Weighted ns.

^b^ The first AUDIT‐C question on the frequency of alcohol consumption acted as a screening for asking additional alcohol‐related questions later in the survey, including the remaining two questions on AUDIT‐C (units consumed per occasion and the frequency of HED). Three hundred and thirty‐seven participants who reported never drinking alcohol in the last year or since COVID‐19 (frequency of drinking = 0 for pre‐ or post‐COVID‐19) were not asked about units consumed or frequency of HED and were assigned 0 for these answers. Missing data is due to attrition from the survey. Also, unlike the question about frequency of drinking occasions, the two questions asking about units and HED occasions also had ‘prefer not to say’ or ‘don't know’ response options which were also treated as missing.

AUDIT‐C, Alcohol Use Disorders Identification Test‐Concise; HED, heavy episodic drinking.

### Associations with increases in the frequency of drinking in lockdown

Amongst women, increases in the frequency of drinking occasions were independently positively associated with more last‐year alcohol reduction attempts, deterioration in psychological wellbeing and physical health and believing that alcohol was unlikely to put them at greater risk of getting or not recovering from COVID‐19 (Table 3). Amongst men, increases in the frequency of drinking were independently positively associated with being younger, having a lower pre‐COVID AUDIT, being furloughed, deterioration in living conditions, deterioration in financial circumstances, deterioration in psychological wellbeing, improvements in social relationships, having fewer pre‐existing health conditions and believing that alcohol was unlikely to put them at greater risk of getting or not recovering from COVID‐19. The pattern of results in the unweighted analysis was broadly similar for women and men, see Table S1 (Supporting Information) for the unweighted model.

**Table 3:** Independent associations of drinking more frequently among men and women – results from fully adjusted binary logistic regression models

	Women^a^			Men^b^		
	OR	95% CI	*P*	OR	95% CI	*P*
Age	0.99	0.98, 1.00	0.113	0.98	0.97, 0.99	**0.001**
Ethnicity	1.27	0.76, 2.11	0.358	0.85	0.49, 1.48	0.573
Education	1.14	0.82, 1.59	0.446	0.97	0.68, 1.40	0.884
Alcohol reduction attempts	1.13	1.06, 1.21	**<0.001**	1.02	0.95, 1.09	0.593
Pre‐COVID‐19 AUDIT	0.98	0.93, 1.03	0.389	0.95	0.91, 1.00	**0.038**
Living alone	0.93	0.61, 1.40	0.711	1.35	0.92, 1.97	0.121
Living with children	1.42	0.99, 2.04	0.058	1.20	0.81, 1.80	0.366
Furloughed	1.25	0.81, 1.91	0.315	2.62	1.61, 4.26	**<0.001**
Keyworker	1.19	0.86, 1.64	0.292	0.90	0.62, 1.30	0.555
Work from home	1.25	0.92, 1.71	0.148	0.78	0.55, 1.11	0.163
Social distancing experience	1.00	0.99, 1.00	0.314	1.00	0.99, 1.00	0.560
*Changes from pre‐ to post‐COVID‐19 in*:						
Living conditions	0.96	0.74, 1.24	0.746	1.52	1.19, 1.95	**0.001**
Financial situation	1.08	0.91, 1.28	0.381	1.24	1.04, 1.49	**0.020**
Psychological wellbeing	1.29	1.11, 1.51	**0.001**	1.27	1.04, 1.54	**0.018**
Social relationships	0.92	0.79, 1.06	0.252	0.74	0.63, 0.87	**<0.001**
Family relationships	1.06	0.91, 1.24	0.450	1.01	0.85, 1.22	0.884
Physical health	1.20	1.01, 1.42	**0.041**	1.11	0.91, 1.36	0.317
Pre‐existing conditions	0.88	0.70, 1.10	0.260	0.77	0.60, 0.99	**0.040**
Perceived alcohol risk	0.99	0.99, 1.00	**0.012**	0.99	0.98, 1.00	**0.001**
Ex‐smoker^c^	1.26	0.92, 1.73	0.157	1.19	0.83, 1.71	0.350
Current smoker	1.09	0.74, 1.60	0.654	1.38	0.93, 2.05	0.105
Change in exercise frequency	0.97	0.93, 1.01	0.175	0.97	0.93, 1.02	0.274

^a^ Weighted *n* = 1030 *X* ^2^(22) = 75.76, *P* < 0.001.

^b^ Weighted *n* = 950, *X* ^2^(22) = 113.16, *P* < 0.001.

^c^ Never smokers as reference category for all smoking variables.

AUDIT‐C, Alcohol Use Disorders Identification Test‐Concise; CI, confidence interval; OR, odds ratio.

### Associations with increases in units consumed per drinking occasion in lockdown

Amongst women, increases in units consumed per drinking occasion were independently positively associated with last‐year alcohol reduction attempts, deterioration in financial circumstances and deterioration in physical health (Table 4). Amongst men, increases in units consumed per drinking occasion were independently positively associated with living with children, deterioration in financial circumstances, deterioration in psychological wellbeing, deterioration in physical health and believing that alcohol was unlikely to put them at greater risk of getting or not recovering from COVID‐19. The unweighted analysis was broadly the same for women and men, with fewer significant predictors for men. See Table S2 (Supporting Information) for unweighted model.

**Table 4:** Independent associations of drinking more units per drinking session among men and women – results from fully adjusted binary logistic regression models

	Women^a^			Men^b^		
	OR	95% CI	*P*	OR	95% CI	*P*
Age	1.00	0.98, 1.01	0.853	1.00	0.99, 1.02	0.966
Ethnicity	2.05	0.96, 4.36	0.063	1.40	0.65, 3.01	0.389
Education	1.30	0.80, 2.11	0.286	0.79	0.51, 1.21	0.276
Alcohol reduction attempts	1.10	1.03, 1.19	**0.009**	1.03	0.95, 1.11	0.503
Pre‐COVID‐19 AUDIT	1.06	0.99, 1.14	0.093	0.97	0.91, 1.03	0.373
Living alone	0.96	0.55, 1.69	0.899	0.77	0.47, 1.26	0.303
Living with children	1.38	0.86, 2.22	0.180	1.72	1.09, 2.73	**0.020**
Furloughed	1.24	0.69, 2.20	0.473	0.98	0.53, 1.80	0.943
Keyworker	1.33	0.87, 2.05	0.191	1.30	0.84, 2.02	0.240
Work from home	1.36	0.89, 2.06	0.153	1.10	0.72, 1.69	0.651
Social distancing experience	1.00	0.99, 1.01	0.699	1.00	0.99, 1.01	0.761
*Changes from pre‐ to post‐COVID‐19 in*:						
Living conditions	1.02	0.73, 1.43	0.892	1.23	0.92, 1.64	0.157
Financial situation	1.31	1.05, 1.64	**0.017**	1.50	1.21, 1.86	**<0.001**
Psychological wellbeing	1.17	0.95, 1.45	0.149	1.35	1.06, 1.73	**0.017**
Social relationships	0.91	0.74, 1.11	0.334	0.88	0.72, 1.07	0.208
Family relationships	1.07	0.87, 1.32	0.536	1.06	0.85, 1.33	0.601
Physical health	1.66	1.31, 2.10	**<0.001**	1.31	1.03, 1.67	**0.027**
Pre‐existing conditions	1.10	0.81, 1.50	0.529	0.93	0.69, 1.25	0.629
Perceived alcohol risk	1.00	0.99, 1.01	0.760	0.99	0.98, 1.00	**0.005**
Ex‐smoker^c^	1.25	0.82, 1.92	0.302	1.04	0.66, 1.63	0.879
Current smoker	0.98	0.57, 1.68	0.950	1.54	0.95, 2.50	0.079
Change in exercise frequency	0.96	0.91, 1.02	0.180	1.00	0.94, 1.06	0.994

^a^ Weighted *n* = 910, *X* ^2^(22) = 74.68, *P* < 0.001.

^b^ *n* = 844, *X* ^2^(22) = 100.43, *P* < 0.001.

^c^ Never smokers as reference category for all smoking variables.

AUDIT‐C, Alcohol Use Disorders Identification Test‐Concise; CI, confidence interval; OR, odds ratio.

### Associations with increases in the frequency of HED in lockdown

Amongst women, increases in the frequency of HED were independently positively associated with being younger, last‐year alcohol reduction attempts, living alone, being furloughed and deterioration in psychological wellbeing (Table 5). Amongst men, increases in the frequency of HED were independently positively associated with living with children, being furloughed, having a more negative experience of social distancing, deterioration in financial circumstances, deterioration in psychological wellbeing, improvements in social relationships and being a current smoker (relative to never smokers). The pattern of unweighted results was broadly similar, see Table S3 (Supporting Information) for unweighted model.

**Table 5:** Independent associations of having more heavy episodic drinking among men and women – results from fully adjusted binary logistic regression models

	Women^a^			Men^b^		
	OR	95% CI	*P*	OR	95% CI	*P*
Age	0.98	0.97, 1.00	**0.027**	0.99	0.98, 1.01	0.475
Ethnicity	0.99	0.50, 1.94	0.964	1.08	0.49, 2.39	0.858
Education	0.64	0.41, 1.01	0.055	1.67	0.98, 2.84	0.060
Alcohol reduction attempts	1.16	1.07, 1.25	**<0.001**	1.00	0.92, 1.09	0.979
Pre‐COVID‐19 AUDIT	1.06	0.98, 1.14	0.134	1.02	0.95, 1.09	0.621
Living alone	1.75	1.04, 2.96	0.**035**	1.67	0.97, 2.87	0.063
Living with children	1.04	0.63, 1.72	0.881	2.40	1.44, 3.99	**0.001**
Furloughed	2.06	1.19, 3.56	**0.010**	3.25	1.80, 5.86	**<0.001**
Keyworker	1.51	0.97, 2.35	0.070	1.51	0.93, 2.46	0.098
Work from home	1.00	0.65, 1.52	0.981	1.14	0.72, 1.80	0.573
Social distancing experience	1.75	1.04, 2.96	0.141	0.99	0.98, 1.00	**0.007**
*Changes from pre‐ to post‐COVID‐19 in*:						
Living conditions	1.00	0.71, 1.41	0.989	0.86	0.63, 1.19	0.363
Financial situation	1.20	0.96, 1.51	0.112	1.58	1.24, 2.02	**<0.001**
Psychological wellbeing	1.46	1.17, 1.82	**0.001**	1.65	1.25, 2.18	**<0.001**
Social relationships	0.85	0.69, 1.04	0.104	0.60	0.47, 0.77	**<0.001**
Family relationships	0.98	0.79, 1.22	0.865	1.29	0.99, 1.69	0.055
Physical health	1.23	0.97, 1.55	0.082	0.98	0.75, 1.30	0.909
Pre‐existing conditions	1.21	0.89, 1.65	0.233	0.81	0.56, 1.18	0.270
Perceived alcohol risk	1.00	0.99, 1.01	0.675	1.00	0.99, 1.01	0.519
Ex‐smoker^c^	1.46	0.94, 2.27	0.090	1.00	0.59, 1.70	0.995
Current smoker	1.26	0.74, 2.13	0.391	2.29	1.33, 3.96	**0.003**
Change in exercise frequency	1.02	0.96, 1.08	0.591	1.00	0.93, 1.07	0.908

^a^ Weighted *n* = 922, *X* ^2^(22) = 89.41, *P* < 0.001.

^b^ Weighted *n* = 848, *X* ^2^(22) = 118.69, *P* < 0.001.

^c^ Never smokers as reference category for all smoking variables.

AUDIT‐C, Alcohol Use Disorders Identification Test‐Concise; CI, confidence interval; OR, odds ratio.

## Discussion

About a one‐third of our self‐selected sample, surveyed between 30 April and 14 June 2020, reported drinking more frequently in the first UK lockdown. These rates were roughly equivalent between men and women though the correlates of increased frequency of alcohol consumption differed somewhat by gender. Deterioration in psychological wellbeing and believing that alcohol was unlikely to put them at greater risk of getting or not recovering from COVID‐19 were correlated with increased frequency of drinking for both men and women. Amongst women, increases in the frequency of drinking occasions were also associated with last‐year alcohol reduction attempts and deterioration in physical health. Whereas amongst men, increases in the frequency of drinking were associated with being younger, having a lower baseline AUDIT‐C score, being furloughed, deterioration in living conditions, deterioration in financial circumstances, improvements in social relationships and having fewer pre‐existing health conditions.

In terms of changes in the units consumed per drinking occasion, women were more likely than men to drink the same number of units as pre COVID‐19 (66% vs. 56% of men). Men were more likely than women to drink both more units (19% vs. 14%) and less units (25% vs. 20%). Deterioration in financial circumstances and physical health were associated with increased unit consumption for both men and women. Amongst women, increases in units consumed per drinking occasion were associated with having more alcohol reduction attempts in the last year. Whereas, amongst men, increases in units consumed per drinking occasion were associated with living with children, deterioration in psychological wellbeing and believing that alcohol was unlikely to put them at greater risk of getting or not recovering from COVID‐19.

Finally, the majority (61%) reported no change in the frequency of HED occasions pre‐ and post‐COVID‐19. For both women and men, being furloughed and deterioration in psychological wellbeing were associated with increases in the frequency of HED. Amongst women, increases in the frequency of HED were also associated with being younger, last‐year alcohol reduction attempts and living alone. Amongst men, increases in the frequency of HED were associated with living with children, having a more negative experience of social distancing, deterioration in financial circumstances, improvements in social relationships and being a current smoker.

### Implications

This study has important implications in terms of highlighting groups that may need targeted support for alcohol reduction to counteract an increase in drinking during future COVID‐19‐related lockdowns in the UK. There were some consistencies in correlates of increased drinking amongst men and women. Deterioration in psychological wellbeing was one of the most consistent predictors of increases in the frequency of drinking and HED for both men and women. Being furloughed was also a consistent predictor of increases in HED across men and women. This is in line with other literature showing that alcohol consumption increases in economic downturns where unemployment is higher, partly due to increases in leisure time, amongst those who are unemployed [36]. These findings suggest that in future iterations of lockdown, those on furlough or similar schemes may require additional alcohol‐related support. Communications from either the government or employers around the furlough scheme could contain links to resources to help individuals manage their wellbeing and their drinking.

In line with other studies [3], there is also some evidence of gender differences in drinking patterns; units consumed per drinking occasion have polarised more amongst men in that men are more likely than women to be drinking both more and less. Furthermore, the correlates of increases in each drinking pattern are different for men and women. Living with children was associated with an increase in units consumed and the frequency of heavy episodic drinking for men, but not women. Increases in the amount of alcohol consumed on drinking occasions amongst male parents are concerning as HED in particular is likely to impair performance of caring responsibilities. This is in line with some research showing that women disproportionately carry the burdens of increased child care [23, 24], which could explain greater declines in wellbeing amongst women [25, 26].

Deterioration in financial circumstances was consistently associated with increases in all of the drinking measures for men, but only with units consumed for women. Previous research examining the relationships between economic downturns and alcohol consumption also found men were more likely to drink heavily in response to recessions and increased unemployment [36]. This may be due to increased stress in response to traditional gender roles in which men may be more likely to be considered the breadwinner.

### Strengths and limitations

A key strength of this study was the variety of measures collected, permitting a detailed analysis of a broad range of potential factors predicting drinking patterns during the start of COVID‐19‐related lockdown in the UK. Furthermore, this survey allowed participants to select the date that they felt COVID‐19 started to affect them, this offers a strength over other studies that rely on using the date lockdown began to signal ‘before’ and ‘after’, in a period of ongoing change. Although the full lockdown began on 24 March, there was advice and knowledge of the virus and its effects in the months before this which may have affected drinking behaviour. For example, pubs in England were closed on 20 March and people were encouraged to work from home or socially distance from 16 March. Furthermore, the collection of data while lockdown was ongoing limits the potential for recall bias, which might be present in retrospective studies. However, this study was not without limitations. As pre‐COVID‐19 drinking is only measured post‐COVID‐19, it may be susceptible to recall bias. The sample was self rather than randomly selected, which reduces the generalisability of these results. Specifically, the results may be more reflective of people who complete online surveys about health than the general population in the UK. Black, Asian and Minority Ethnic people in particular were underrepresented in the study sample, this means that ethnicity was treated as white versus minority ethnic groups. Grouping all Black, Asian and Minority Ethnic participants together in this way does not allow examination of differences between different ethnicities and cultures, which limits the generalisability of these conclusions further. Finally, here we use a non‐validated measure of self‐assessed changes in psychological wellbeing, which may have been interpreted differently by participants.

## Conclusions

Thirty percent of participants reported drinking more frequently in lockdown, 16% reported drinking more units per drinking occasion and 14% reported more frequent heavy episodic drinking. Men were more likely than women to drink both more and fewer units per drinking occasion, whereas shifts in other drinking patterns were roughly equivalent. There were some correlates of increased drinking patterns that were consistent across men and women; being less likely to believe alcohol drinking would lead to greater chance of catching COVID‐19 or worse outcomes, and deterioration in psychological wellbeing was consistently associated with increased frequency of drinking. Deterioration in financial situation and physical health were associated with increased unit consumption and deterioration in psychological wellbeing and being furloughed consistently predicted increases in heavy episodic drinking. Other gender differences were detected in the correlates of increased drinking. This study identifies groups that may require targeted support in the event of future lockdowns. However, this sample was predominantly white and further research examining the effect of lockdown on drinking amongst a more representative sample of UK drinkers would be of value.

## Conflict of Interest

MO, CG, JB, DK, AH and LS declare no conflicts of interest. JB and LS have received unrestricted research grants from Pfizer related to smoking cessation.

## Ethics Statement

The study has been approved by UCL Research Ethics Committee at the UCL Division of Psychology and Language Sciences (CEHP/2020/579) as part of the larger program ‘The optimisation and implementation of interventions to change behaviours related to health and the environment’. All participants provided fully informed consent. The study is General Data Protection Regulation compliant.

## Supporting information


**Appendix S1.** Full description of measures.
**Appendix S2.** Unweighted associations.
