# Supplementary material for: Characterising the patterns of and factors associated with increased alcohol consumption since COVID‐19 in a UK sample
Source: Drug Alcohol Rev. 2021 Mar 3;40(6):890–9. doi: 10.1111/dar.13256 (PMC8014505; doi:10.1111/dar.13256)
Supplement: Supplementary file 1 — Appendix S1. Full description of measures. Appendix S2. Unweighted associations. [file DAR-40-890-s001.docx]

**Appendix S1: Full description of measures**

**Outcome Variables**

*Increase in frequency of drinking*

Frequency of drinking occasions was measured by the question “How often did you have a drink containing alcohol in the past year/since COVID?” with the response options ‘never’, ‘monthly or less’, ‘two to four times a month’, ‘two to three times per week’ or ‘four times a week’. Participants who reported drinking more frequently post COVID-19 than in the 12 months prior to COVID by one category or more were coded as 1 relative to those who reported drinking less or the same.

*Increase in units consumed*

Units consumed per drinking occasion was measured by the question “How many units of alcohol did you have on a typical day when you were drinking in the past year/since COVID?” with the response options ‘1 or 2 units’, ‘3 or 4 units’, ‘5 or 6 units’, ‘7 to 9 units’ or ‘10 or more units’. Participants who reported drinking more units post COVID-19 than in the 12 months prior to COVID by one category or more were coded as 1 relative to those who reported drinking less or the same.

*Increase in heavy episodic drinking*

Frequency of heavy episodic drinking was measured by the question “How often did you have six or more drinks on one occasion in the past year/since COVID?” with the response options ‘never’, ‘less than monthly’, ‘monthly’, ‘weekly’, ‘daily’ or ‘almost daily’. Participants who reported more frequent heavy episodic drinking post COVID-19 than in the 12 months prior to COVID by one category or more were coded as 1 relative to those who reported drinking less or the same.

**Predictor Variables**

*Sociodemographic factors*

The following sociodemographic characteristics were included: age (continuous in years 18-110+), ethnicity (white = 1, Black, Asian and Minority Ethnic = 0; due to a small proportion of respondents from the individual ethnic minorities, which is a limitation of the study) and education (with post-16 qualifications = 1, other = 0). Post 16 education are optional qualifications attained above the age of 16, e.g. A-levels, Scottish Highers, university degrees, vocational training and work-based learning. Education provides a reliable indication of socioeconomic position prior to COVID-19 as it is not affected by recent job loss or furlough. Previous research has also shown that level of education is one of the strongest socioeconomic predictors of volume of alcohol consumption in England [35].

*Baseline drinking characteristics*

Alcohol reduction attempts in last 12 months

Measured by the question “How many attempts to reduce how much alcohol you drink have you made in the last 12 months (e.g. by drinking less, choosing lower strength alcohol, using smaller glasses, or in some other way)? Include all attempts, including unsuccessful and current attempts.” Treated as a continuous variable from 0-14+. All responses above 13 were grouped into a single response category of 14 and over.

Pre-COVID Alcohol Use Disorders Identification Test-Concise score

Total across the three questions regarding frequency of drinking, units consumed and frequency of heavy episodic drinking as outlined above at baseline (e.g. in the 12 months pre COVID-19). Continuous score between 0-12.

*Living situation*

Living alone

Measured with question “How many persons other than yourself (including children) live with you now in the same flat or house?”; Coded as 1= live alone, 0 = live with others).

Living with children

Measured with the question “Do you live with any of these persons below?” (select all that apply) with the answers ‘with my partner’, ‘husband/wife’, ‘boyfriend/girlfriend’, ‘with children 0-5 years old’, ‘with children 6-15 years old’, ‘with persons aged 16-69 (family or friends)’, ‘with persons aged 70+ (family or friends)’, ‘persons who you believe may be vulnerable to COVID-19 for any reason’, ‘persons who are in poor health’. Living with children aged <16 coded as 1.

*Furloughed*

Measured by question “What is your current main occupation during COVID-19?”, with the response options ‘employed (full or part time)’, ‘self-employed’, ‘student’, ‘furloughed’, ‘laid off during COVID-19’, ‘unemployed since before COVID-19’, ‘retired’, ‘homemaker, full time parent or carer’, ‘unable to work due to disability’ and ‘other’. Participants reporting being furloughed will be coded as 1, with all other responses coded as 0.

*Keyworker*

Measured with question “Are you currently fulfilling any of the governments identified ‘key worker’ roles (e.g. working in health or social work, public safety, transport, food chain worker etc.); 1= yes, 0 = no. Only those reporting that they were employed or self-employed to the question “What is your current main occupation during COVID-19?” were asked this question and as such all those responding with ‘student’, ‘furloughed’, ‘laid off during COVID-19’, ‘unemployed since before COVID-19’, ‘retired’, ‘homemaker, full time parent or carer’, ‘unable to work due to disability’ and ‘other’ were also coded as 0.

*Work from home*

Measured by question “Can you do your work or study from home?” with responses as ‘yes, I can do all the work or study from home’, ‘I can only do some of my work or study from home’, and ‘no my work or study cannot be done from home’. Those reporting doing some or all their work/study from home were coded as 1, those unable to work from home coded as 0. Only those responding they were employed, self-employed or a student to the question “What is your current main occupation during COVID-19?” were asked this question and as such all those responding with, ‘furloughed’, ‘laid off during COVID-19’, ‘unemployed since before COVID-19’, ‘retired’, ‘homemaker, full time parent or carer’, ‘unable to work due to disability’ and ‘other’ were also coded as 0.

*Experience of social distancing*

Measured by the question “How would you rate your overall experience with social distancing restrictions due to COVID-19” measured on a scale from ‘extremely negative (1)’ to ‘extremely positive (100)’.

*Changes to lifestyle as result of COVID-19*

Change in: living conditions, financial situation, psychological wellbeing, social relationships, family relationships and physical health

Participants were asked to rate each of these areas of their life pre and post COVID on a scale of ‘1 (poor)’ to ‘5 (excellent)’. Change scores were calculated - a negative score indicates an improvement in that domain.

*Health concerns*

Number of pre-existing health conditions

Measured by question “Did a Doctor or health professional ever tell you that you had any of the following conditions? ‘heart disease’, ‘stroke’, high blood pressure or hypertension’, ‘diabetes’, ‘dementia’, ‘liver disease’, ‘cancer (in last 5 years)’, ‘kidney disease’, ‘lung disease (asthma or COPD)’, ‘organ transplant, ‘other conditions leading to immunosuppression (e.g. HIV)’, ‘none of the above’, prefer not to say’. The number of reported existing is summed.

Risk of alcohol for COVID-19

Measured by question ‘I believe that drinking alcohol puts me at a greater risk of getting COVID-19 or not recovering from it’ measured on a scale of ’1 (completely disagree’ to ‘100 completely agree’.

*Other behaviours*

Smoking status

In the pre-registered protocol we planned to have four smoking variables denoting current smoker, never smoker, recent (< 1 month) ex-smoker and long term (> 1 month) ex smoker. Due to low prevalence of recent ex-smokers (n = 12), this variable was combined with long term ex-smokers. The three smoking variables were entered into the model as a categorial variable with never smokers as the reference.

Change in exercise frequency

Participants were asked how many days per week they did strength training (e.g. Pilates, yoga, squats) with the options ‘0 days’, ‘1 day’, ‘2 days’, ‘3 days’ and ‘4 days or more’ both pre and post COVID-19. Participants were asked how many times per week pre/post COVID-19 they did at least 15 minutes or more of moderate or vigorous aerobic physical training (e.g. brisk walk, jogging, dancing) with the response options ranging from 1-14+ times. Both ‘4 or more’ and ‘14+’ will be treated as meaning 4 or 14. A total number of sessions (the number of days of strength training and aerobic sessions combined) both pre and post COVID-19 was summed and a change score calculated.

**Appendix S2. Unweighted associations**

Table S1. Unweighted independent correlates/associations of drinking more frequent among men and women – results from fully adjusted binary logistic regression models.

|  | **Women^1^** | | | | **Men^2^** | | | |
| --- | --- | --- | --- | --- | --- | --- | --- | --- |
|  | OR | 95% CI | | *P* | OR | 95% CI | | *P* |
| Age | **0.99** | **0.98, 1.00** | | **0.005** | **0.98** | **0.97, 0.99** | | **0.001** |
| Ethnicity | 1.62 | 0.96, 2.73 | | 0.069 | 1.42 | 0.66, 3.05 | | 0.369 |
| Education | 1.30 | 0.89, 1.91 | | 0.173 | 0.98 | 0.57, 1.66 | | 0.930 |
| Alcohol reduction attempts | **1.11** | **1.05, 1.17** | | **0.000** | 1.07 | 0.99, 1.15 | | 0.115 |
| Pre-COVID-19 AUDIT | 0.97 | 0.93, 1.02 | | 0.217 | **0.92** | **0.87, 0.98** | | **0.005** |
| Living alone | 0.97 | 0.71, 1.33 | | 0.852 | 1.45 | 0.94, 2.23 | | 0.094 |
| Living with children | **1.51** | **1.15, 1.99** | | **0.003** | 1.18 | 0.77, 1.80 | | 0.454 |
| Furloughed | 1.14 | 0.78, 1.67 | | 0.499 | **2.47** | **1.36, 4.50** | | **0.003** |
| Keyworker | 1.21 | 0.94, 1.55 | | 0.135 | 1.21 | 0.81, 1.80 | | 0.357 |
| Work from home | 1.15 | 0.90, 1.46 | | 0.268 | 1.04 | 0.71, 1.53 | | 0.824 |
| Social distancing experience | 1.00 | 1.00, 1.01 | | 0.624 | 1.00 | 0.99, 1.01 | | 0.839 |
| *Changes from pre- to post-COVID-19 in*: | | |  |  |  |  |  |  |
| Living conditions | 0.96 | 0.79, 1.17 | | 0.687 | 1.23 | 0.94, 1.62 | | 0.131 |
| Financial situation | 1.05 | 0.92, 1.20 | | 0.431 | 1.21 | 0.99, 1.47 | | 0.057 |
| Psychological wellbeing | **1.32** | **1.16, 1.50** | | **0.000** | 1.07 | 0.87, 1.31 | | 0.555 |
| Social relationships | 0.93 | 0.82, 1.05 | | 0.246 | 0.84 | 0.70, 1.02 | | 0.077 |
| Family relationships | 1.03 | 0.91, 1.17 | | 0.670 | 1.11 | 0.90, 1.38 | | 0.326 |
| Physical health | **1.24** | **1.08, 1.43** | | **0.002** | 1.18 | 0.94, 1.49 | | 0.164 |
| Pre-existing conditions | 0.99 | 0.83, 1.19 | | 0.911 | 0.83 | 0.63, 1.10 | | 0.190 |
| Perceived alcohol risk | **0.99** | **0.99, 1.00** | | **0.003** | **0.99** | **0.99, 1.00** | | **0.037** |
| Ex-smoker^3^ | 1.27 | 0.92, 1.76 | | 0.152 | 1.26 | 0.80, 2.00 | | 0.319 |
| Current smoker | 1.06 | 0.82, 1.36 | | 0.664 | 1.33 | 0.90, 1.96 | | 0.155 |
| Change in exercise frequency | 0.98 | 0.95, 1.01 | | 0.261 | 0.98 | 0.93, 1.04 | | 0.527 |

^1^N = 1584 X^2^(22) = 109.45, *P* <0.001, ^2^N = 713, X^2^(22) = 64.26, *P* <0.001, ^3^Never smokers as reference category for all smoking variables. AUDIT, Alcohol Use Disorders Identification Test; CI, confidence interval; OR, odds ratio.

Table S2. Unweighted independent correlates/associations of drinking more units per drinking session among men and women – results from fully adjusted binary logistic regression models

|  | **Women^1^** | | | **Men^2^** | | | |
| --- | --- | --- | --- | --- | --- | --- | --- |
|  | OR | 95% CI | *P* | OR | 95% CI | | *P* |
| Age | 1.00 | 0.99, 1.01 | 0.587 | 1.00 | 0.99, 1.02 | | 0.899 |
| Ethnicity | 2.09 | 0.95, 4.60 | 0.067 | 1.06 | 0.42, 2.65 | | 0.909 |
| Education | 1.24 | 0.72, 2.12 | 0.439 | 0.77 | 0.42, 1.42 | | 0.407 |
| Alcohol reduction attempts | **1.11** | **1.05, 1.17** | **0.000** | 1.00 | 0.91, 1.09 | | 0.947 |
| Pre-COVID-19 AUDIT | 1.05 | 0.99, 1.11 | 0.094 | 0.97 | 0.90, 1.04 | | 0.335 |
| Living alone | 1.14 | 0.75, 1.74 | 0.543 | 1.09 | 0.64, 1.85 | | 0.746 |
| Living with children | **1.54** | **1.08, 2.19** | **0.016** | **1.72** | **1.06, 2.78** | | **0.028** |
| Furloughed | 1.32 | 0.80, 2.16 | 0.278 | 1.17 | 0.58, 2.34 | | 0.657 |
| Keyworker | 1.34 | 0.97, 1.85 | 0.079 | 1.36 | 0.85, 2.18 | | 0.197 |
| Work from home | 1.20 | 0.87, 1.66 | 0.258 | 1.25 | 0.80, 1.96 | | 0.322 |
| Social distancing experience | 1.01 | 1.00, 1.01 | 0.052 | 1.00 | 0.99, 1.01 | | 0.868 |
| *Changes from pre- to post-COVID-19 in:* | | |  |  |  |  |  |
| Living conditions | 1.18 | 0.92, 1.52 | 0.190 | 0.99 | 0.72, 1.36 | | 0.957 |
| Financial situation | **1.27** | **1.07, 1.51** | **0.006** | **1.37** | **1.09, 1.72** | | **0.007** |
| Psychological wellbeing | **1.27** | **1.07, 1.50** | **0.005** | 1.21 | 0.94, 1.56 | | 0.149 |
| Social relationships | 0.98 | 0.84, 1.16 | 0.848 | 0.92 | 0.73, 1.15 | | 0.449 |
| Family relationships | 1.07 | 0.91, 1.27 | 0.416 | 1.14 | 0.88, 1.46 | | 0.324 |
| Physical health | **1.46** | **1.21, 1.75** | **0.000** | 1.15 | 0.87, 1.51 | | 0.321 |
| Pre-existing conditions | 1.06 | 0.84, 1.35 | 0.621 | 0.94 | 0.68, 1.29 | | 0.690 |
| Perceived alcohol risk | 1.00 | 0.99, 1.00 | 0.658 | 1.00 | 0.99, 1.01 | | 0.622 |
| Ex-smoker^3^ | 1.16 | 0.75, 1.79 | 0.505 | **2.27** | **1.32, 3.92** | | **0.003** |
| Current smoker | 1.33 | 0.96, 1.85 | 0.086 | 1.51 | 0.94, 2.42 | | 0.090 |
| Change in exercise frequency | 0.97 | 0.93, 1.02 | 0.201 | 1.05 | 0.99, 1.12 | | 0.123 |

^1^ N = 1449, X^2^(22) = 108.12, P <0.001, ^2^N = 655, X^2^(22) = 50.77, *P* <0.001, ^3^Never smokers as reference category for all smoking variables. AUDIT, Alcohol Use Disorders Identification Test; CI, confidence interval; OR, odds ratio.

Table S3. Unweighted independent correlates/associations of having more heavy episodic drinking among men and women – results from fully adjusted binary logistic regression models

|  | **Women^1^** | | | | **Men^2^** | | | |
| --- | --- | --- | --- | --- | --- | --- | --- | --- |
|  | OR | 95% CI | | *P* | OR | 95% CI | | *P* |
| Age | **0.98** | **0.97, 0.99** | | **0.004** | 0.99 | 0.97, 1.00 | | 0.082 |
| Ethnicity | 1.92 | 0.87, 4.21 | | 0.105 | 1.45 | 0.50, 4.17 | | 0.493 |
| Education | 0.93 | 0.54, 1.59 | | 0.781 | 1.61 | 0.77, 3.39 | | 0.206 |
| Alcohol reduction attempts | **1.13** | **1.07, 1.20** | | **<0.001** | 1.06 | 0.97, 1.17 | | 0.210 |
| Pre-COVID-19 AUDIT | 1.04 | 0.98, 1.10 | | 0.207 | 0.96 | 0.89, 1.03 | | 0.235 |
| Living alone | 1.25 | 0.81, 1.94 | | 0.319 | 1.29 | 0.73, 2.28 | | 0.374 |
| Living with children | 1.24 | 0.86, 1.79 | | 0.251 | 1.83 | 1.10, 3.03 | | 0.019 |
| Furloughed | **1.94** | **1.20, 3.14** | | **0.007** | 2.73 | **1.38, 5.40** | | **0.004** |
| Keyworker | **1.61** | **1.15, 2.26** | | **0.005** | 2.06 | **1.27, 3.33** | | **0.003** |
| Work from home | 1.02 | 0.73, 1.42 | | 0.931 | 1.11 | 0.69, 1.77 | | 0.670 |
| Social distancing experience | 1.00 | 0.99, 1.01 | | 0.549 | 1.00 | 0.99, 1.01 | | 0.613 |
| *Changes from pre- to post-COVID-19 in:* | | |  |  |  |  |  |  |
| Living conditions | 1.13 | 0.87, 1.47 | | 0.346 | 0.95 | 0.68, 1.34 | | 0.785 |
| Financial situation | 1.24 | 1.04, 1.48 | | 0.015 | **1.32** | **1.04, 1.69** | | **0.024** |
| Psychological wellbeing | **1.41** | **1.19, 1.68** | | **<0.001** | 1.24 | 0.95, 1.63 | | 0.119 |
| Social relationships | 0.87 | 0.74, 1.03 | | 0.116 | **0.76** | **0.59, 0.98** | | **0.033** |
| Family relationships | 0.96 | 0.81, 1.15 | | 0.682 | 1.28 | 0.97, 1.68 | | 0.083 |
| Physical health | **1.26** | **1.04, 1.52** | | **0.017** | 1.07 | 0.80, 1.43 | | 0.639 |
| Pre-existing conditions | 1.21 | 0.95, 1.55 | | 0.121 | 1.04 | 0.73, 1.47 | | 0.840 |
| Perceived alcohol risk | 1.00 | 1.00, 1.01 | | 0.731 | 1.00 | 1.00, 1.01 | | 0.492 |
| Ex-smoker^3^ | 1.13 | 0.72, 1.76 | | 0.593 | **2.02** | **1.13, 3.62** | | **0.018** |
| Current smoker | 1.27 | 0.90, 1.79 | | 0.178 | 1.43 | 0.86, 2.37 | | 0.169 |
| Change in exercise frequency | 0.99 | 0.94, 1.03 | | 0.585 | 1.05 | 0.98, 1.13 | | 0.146 |

^1^N = 1451, X^2^(22) = 108.01, *P* <0.001, ^2^N = 656, X^2^(22) = 67.40, *P* <0.001, ^3^Never smokers as reference category for all smoking variables. AUDIT, Alcohol Use Disorders Identification Test; CI, confidence interval; OR, odds ratio.
